# Applying the theory of planned behavior to self-report dental attendance in Norwegian adults through structural equation modelling approach

**DOI:** 10.1186/s12903-018-0558-7

**Published:** 2018-05-31

**Authors:** Anne N. Åstrøm, Stein Atle Lie, Ferda Gülcan

**Affiliations:** 1 Oral health Centre of Expertise in Western Norway, Bergen, Hordaland Norway; 2 Department of Clinical Dentistry, Faculty of Medicine, University of Bergen, PO Box 7804, N-5020 Bergen, Norway

**Keywords:** Dental attendance, Young adults, Theory of planned behavior, Structural equation modelling, AMOS

## Abstract

**Background:**

Understanding factors that affect dental attendance behavior helps in constructing effective oral health campaigns. A socio-cognitive model that adequately explains variance in regular dental attendance has yet to be validated among younger adults in Norway. Focusing a representative sample of younger Norwegian adults, this cross-sectional study provided an empirical test of the Theory of Planned Behavior (TPB) augmented with descriptive norm and action planning and estimated direct and indirect effects of attitudes, subjective norms, descriptive norms, perceived behavioral control and action planning on intended and self-reported regular dental attendance.

**Method:**

Self-administered questionnaires provided by 2551, 25–35 year olds, randomly selected from the Norwegian national population registry were used to assess socio-demographic factors, dental attendance as well as the constructs of the augmented TPB model (attitudes, subjective norms, descriptive norms, intention, action planning). A two-stage process of structural equation modelling (SEM) was used to test the augmented TPB model.

**Results:**

Confirmatory factor analysis, CFA, confirmed the proposed correlated 6-factor measurement model after re-specification. SEM revealed that attitudes, perceived behavioral control, subjective norms and descriptive norms explained intention. The corresponding standardized regression coefficients were respectively (β = 0.70), (β =0.18), (β = − 0.17) and (β =0.11) (*p* < 0.001). Intention (β =0.46) predicted action planning and action planning (β =0.19) predicted dental attendance behavior (*p* < 0.001). The model revealed indirect effects of intention and perceived behavioral control on behavior through action planning and through intention and action planning, respectively. The final model explained 64 and 41% of the total variance in intention and dental attendance behavior.

**Conclusion:**

The findings support the utility of the TPB, the expanded normative component and action planning in predicting younger adults’ intended- and self-reported dental attendance. Interventions targeting young adults’ dental attendance might usefully focus on positive consequences following this behavior accompanied with modeling and group performance.

**Electronic supplementary material:**

The online version of this article (10.1186/s12903-018-0558-7) contains supplementary material, which is available to authorized users.

## Background

The importance of dental attendance is a key measure in health education as regular attendance is associated with good health and well-being. In Norway, the Public Dental Service (PDS) is financed by taxes and provides free dental care to children and adolescents until 20 years of age [1, 2]. The private dental services provides dental care to the general adult population and is organized according to market mechanisms, with dental fees determined by supply and demand and with very limited private or public insurance arrangements [3]. Regardless of the disparities in dental coverage between Norway and other Scandinavian countries, dental attendance rates have been high among Norwegian adults. About 80 and 77% of Norwegian adults above 20 years of age reported having visited a dentist during the last 12 months in 2008 and 2013, respectively [4–6].

Nevertheless, as in other Scandinavian countries, the prevalence of regular dental care utilization among Norwegian adults varies according to age, period and socio-economic status, being smallest in the younger age- and the lower income groups [4–7]. Støle et al. [8] found that 87% of 23–24 year old Norwegian adults visited a dentist every second year in 1983, whereas the corresponding figures in 1994 was 85%. Among 25 year old Norwegian adults, 62 and 44% reported dental attendance once a year in 1997 and 2007, respectively [9]. According to the Official Statistics of Norway, the prevalence of having visited public dental health care services during last year was highest among 45–66 year olds and lowest among 21–24 year olds in 2016 [10]. Previous studies have identified enabling factors, such as cost of treatment and dental anxiety, as important barriers towards regular use of dental care [11, 12]. In a recent population-based study of Swedish adults, financial problems and lack of social support were associated with refraining from seeking dental care [11]. Whereas socio-demographic- and need related factors are important covariates of dental care utilization, relatively few studies have considered modifiable socio-cognitive determinants in the younger adult populations. Influencing younger adults’ adherence to continued dental attendance requires understanding of the socio-cognitive factors underlying their decision to comply with advices for regular dental care. A socio-cognitive model that adequately explains variance in regular dental attendance has yet to be validated among younger adults in Norway.

### Theoretical approach

The theory of planned behavior, TPB, is a widely applied socio-cognitive model of the attitude–behavior relationship, assuming that most conscious behaviors is rational and goal directed [13, 14]. TPB proposes a causal link between attitudes and behavior mediated by behavioral intentions. Intention directly influences behavior and is shaped by attitudes, subjective norms and perceived behavioral control regarding the behavior. Empirical validations of the TPB have revealed that the model reliably explains 40–50% of the variance in intention and that intention explains between 20 and 40% of the variance in actual behavior [14–16].

In spite of its predictive success, TPB has been criticized for its validity and it has been shown that other variables explain considerable proportions of the variance in intention and behavior [17]. Moreover, descriptive norms and action planning have shown residual effects on intention and behaviour after consideration of the original TPB variables [17, 18]. Evidence suggests that adding action planning would improve the prediction of behavior from the TPB [18, 19]. Thus, formation of action plans can be used to promote the realization of desired outcomes [18, 19]. The role of subjective norms within the TPB has also been considered [20, 21]. Subjective norms have been criticized for its narrow conceptualization, focusing what significant people thinks others ought to do, neglecting descriptive norms referring to what significant others themselves actually do [20–22]. Descriptive norms correlate with behavioral intentions and have shown to be among the strongest correlates of physical activity [23]. The TPB has received considerable empirical support across health- and social behaviors, including oral hygiene behaviors and health screening [15]. However, to our knowledge, only one previous study has examined use of public dental services in the context of TPB [16].

Focusing a representative sample of young Norwegian adults 25–35 years old, this study provides an empirical test of the TPB augmented with descriptive norm and action planning and estimates direct and indirect effects of attitudes, subjective norms, descriptive norms, perceived control, and action planning on intended and self-reported regular dental attendance. Based on the conceptualization of the TPB and previous empirical support it was hypothesized that the responses to 16 observed indicator variables could be explained by 6 latent factors in terms of attitudes, subjective norms, descriptive norms, perceived control, intention and action planning. Further, it was hypothesized that each indicator would have a stronger relation with their corresponding factor than with the competing factors. Finally, it was hypothesized that attitudes, subjective norm, descriptive norm and perceived behavioral control would predict behavioral intention and that intention, action planning and perceived behavioral control, would predict self-reported dental attendance.

## Methods

### Study design, participants and ethical issues

The present study used data from an electronic, cross-sectional public dental health survey conducted in Norway. A representative sample of 9000 adults (using individuals as the primary sampling unit) aged 25–35 years was randomly selected from the Norwegian national population registry in September 2016. Participation was voluntary and anonymous and the return of a completed questionnaire recognized as the informed consent. Ethical permission to carry out the survey was granted by the Ombudsman, Norwegian Center for Research Data (NO.*49241)*. NORSTAT (www.norstat.no) was responsible for the electronically distributed questionnaires and for the data collection. An eligible sample of 9052 adults aged 25–35 years of age received an electronic version of the questionnaire with an introductory letter explaining the purpose of the study. Total response rate was 29% (2635/9052). Eighty-four respondents were removed due to incomplete questionnaires. All participants who provided complete questionnaires were included in the present study (*n* = 2551).

### Measures

Dental attendance behavior was measured using one question; “How often do you usually visit a dentist?” the response categories ranged from (1) twice a year or more to (4) more seldom than every second year. Components of an augmented version of Ajzen’s TPB [13] was measured in terms of attitudes, subjective norms, perceived behavioral control, descriptive norm and action planning in relation to regular dental attendance. In accordance with recommendations from Ajzen [13], each construct was measured considering the four elements of action (attending), target (dentist), context (on a regular basis), and time (future) (13) (. Intention to attend a dentist regularly was measured by two items, e.g. “I intend to attend a dentist regularly in the future.” Responses were indicated on a four-point scale: (1) Strongly disagree, (2) Disagree, (3) Neither agree nor disagree (4) Agree and (4) Strongly agree. Attitude towards regular dental attendance was assessed by four items, e.g. “to attend a dentist regularly in the future do not make sense to me”. Responses were indicated on a five-point scale ranging from 1 (strongly disagree) to 5 strongly disagree). Subjective norm was measured by three items, e.g. “My parents (partner/friend, dentist) want me to attend a dentist regularly in the future”. Responses were indicated on a five-point scale ranging from 1 (strongly disagree) to 5 (strongly agree). Perceived behavioral control was measured by two items, e.g. “Its totally up to me whether I attend a dentist regularly in the future”. Responses were indicated on a five-point scale ranging from 1 (strongly disagree) to 5 (strongly agree). Descriptive norm was measured by two items, e.g. “My friends attend dentist regularly” – with response categories ranging from 1 (strongly disagree) to 5 (strongly agree). Action planning was assessed using the action planning scale adopted from Sniehotta et al. [17], including three items, e.g. “I have made a detailed plan when to attend, where to attend and how to attend a dentist regularly in the future”. Response categories ranged from 1 (strongly disagree) to 5 (strongly agree). Parts of the questionnaire used in the present study is available in English in the Additional file 1.

### Statistical analyses

Data were analyzed using SPSS version 22.0 (IBM Corp. Released 2013, IBM SPSS Statistics for Windows, Armonk NY: IBM Corp). IBM SPSS AMOS 16.0 [24] was used to test the hypothesized augmented TPB model using a two-step modelling approach whereby the measurement model (step1) and the structural model (step 2) were constructed separately [25]. First, a confirmatory factor analysis, CFA, using maximum likelihood estimation (ML) was conducted to test the adequacy of the measurement model [25]. Modification indices (MI) were used to identify sources of misfit in the model. A prerequisite for testing of invariance across structural paths in the full structural model (step 2) is that the measurement model has configural and metric invariance. Configural invariance was examined by testing the fit of the modified correlated measurement model separately for males and females and by testing the fit of an un-constrained multi-group model. Metric invariance was examined by comparing a multi-group model with all factor loadings constrained equal to the baseline configural model in which the factor loadings were free to vary. The models were assumed non-invariant if the change in chi square was significant and the decrease in comparative fit index, CFI, was less than 0.001 [26].

A full structural equation modelling, SEM, (step 2) examined whether the hypothesized TPB model was acceptable fit to the present data, testing simultaneously the interrelationships specified within the a priori augmented TPB model. To assess how adequately the hypothesized model described the sample data, chi-square test was used together with the following goodness of fit indices; CFI (Comparative fit index), RMSEA (root mean square error of approximation) and AIC (Akaike’s information criteria). In line with the conventional recommendations of Hu and Bentler [27], a good model fit was indicated by a RMSEA less or equal to 0.06, a CFI greater or equal to 0.90 and with a model having lower AIC being the more plausible together with a non-significant Chi square. Statistical significance of parameter estimates are the Critical Ration (CR) representing the parameter estimate divided by its standard error. Based on a level of 0.05, the test statistics (CR) needed to be 1.96 before rejection of the null hypothesis.

## Results

### Sample profile

In spite of the relatively low response rate (29%) obtained, the age distribution of the final sample corresponded with that of the Norwegian population 20–44 years old by December 2016. The age distribution of younger (25–29 years) and older (30–35 years) participants were 43.7 and 56.3%, respectively. Corresponding figures in the population were respectively 46.3 and 53%. Whereas the gender distribution in the sample was 43% men and 56.7% women, the corresponding population distribution was 51.3 and 49.0%. Among the participants, 27.3, 38.6 and 34.1% reported respectively, primary-, bachelor- and college/university level of education. Corresponding figures in the adult population 16 years and above were 26.5, 37.8 and 32.9%. Among the respondents (*n* = 2551), 91.5% were of native Norwegian origin. Eight percent confirmed dental attendance at least twice a year, 47.2% once a year, 21.2% every second year and 21.2% more seldom than every second year (Table 1).

**Table 1:** Frequency distribution of participants’ socio-demographic characteristics and dental attendance behavior, (*n* = 2551) and corresponding percentage figures in the total population

		Participants	Total population
	Category	% (*n*)	%
Gender	Male	43.3 (1105)	51.3
	Female	56.7 (1446)	49.0^a^
Age	25-29 years	43.7 (1116)	46.3
	30–35 years	56.3 (1435)	53.0^a^
Country of birth	Norway	91.5 (2333)	
	Other Nordic	2.6 (66)	
	Outside Nordic	6.0 (152)	
Civil status	Single	36.6 (935)	
	Married	63.4 (1616)	
Highest level of education	Primary/secondary	27.3 (679)	26.5
	Bachelor degree	38.6 (962)	37.8
	College/university	34.1 (850)	32.9^b^
Income (NOK)	At least 400.000	19.5 (413)	
	400,001–800,000	41.2 (876)	
	> 800,000	39.3 (835)	
Dental attendance	At least twice a year	8.0 (203)	
	Once a year	47.2 (1205)	
	Every second year	21.2 (540)	
	More seldom	21.2 (540)	

^a^Norwegian population 20–44 years by December 2016

^b^Norwegian population above 20 years by December 2016

### Descriptive statistics of TPB variables

Table 2 depicts mean, standard deviation, minimum and maximum scores and theoretical range for each indicator measuring the latent constructs of attitudes, subjective norms, perceived control, descriptive norms, intention and action planning. On average the study group demonstrated strong intentions with mean values in the range 4.2–4.3, both positive and negative attitudes (mean values 2.4–4.7), moderate to strong subjective norms (mean values 3.9–4.4), moderate descriptive norms (mean values 3.4–3.9), strong perceived behavioral control (mean values 4.4–4.5), and weak action planning (mean values 2.0–2.2).

**Table 2:** Descriptive statistics of all variables related to the augmented model of planned behavior

	Mean	SD	Min	Max	Theoretical range
Intention					
I intend to attend dentist regularly (Q31_1)	4.3	1.0	1	5	Low-high
I have made a decision to attend (Q31_2)	4.2	1.1	1	5	Low-high
Attitudes					
To attend dentist regularly is:					
---reasonable (Q31_4)	4.7	0.7	1	5	Negative-positive
--necessary (Q31_6)	4.3	1.0	1	5	Negative-positive
-economic burden (Q31_5)	2.4	1.3	1	5	Negative-positive
Intolerable Q31_3	4.2	1.1	1	5	Negative-positive
Subjective norm					
My parents want me to attend regularly (Q31_7)	4.0	1.1	1	5	Low-high
My partner want me to attend regularly (Q31_8)	3.9	1.1	1	5	Low-high
My dentist want me to attend regularly (Q31_9)	4.4	0.9	1	5	Low-high
Descriptive norm					
My friends attend regularly (Q31_12)	3.4	1.0	1	5	Low-high
My parents attend regularly (Q31_13)	3.9	1.1	1	5	Low-high
Perceived control					
It’s up to me to attend regularly (Q31_10)	4.5	0.8	1	5	Low-high
I am capable to attend regularly (Q31_11)	4.4	1.0	1	5	Low-high
Action planning					
I have made a detailed plan regarding--------					
When attending (Q31_14)	2.0	1.2	1	5	Low-high
Where attending (Q31_15)	2.2	1.4	1	5	Low -high
How attending (Q31_16)	2.0	1.3	1	5	Low-high

### Evaluation of the measurement model

**Fig. 1:**
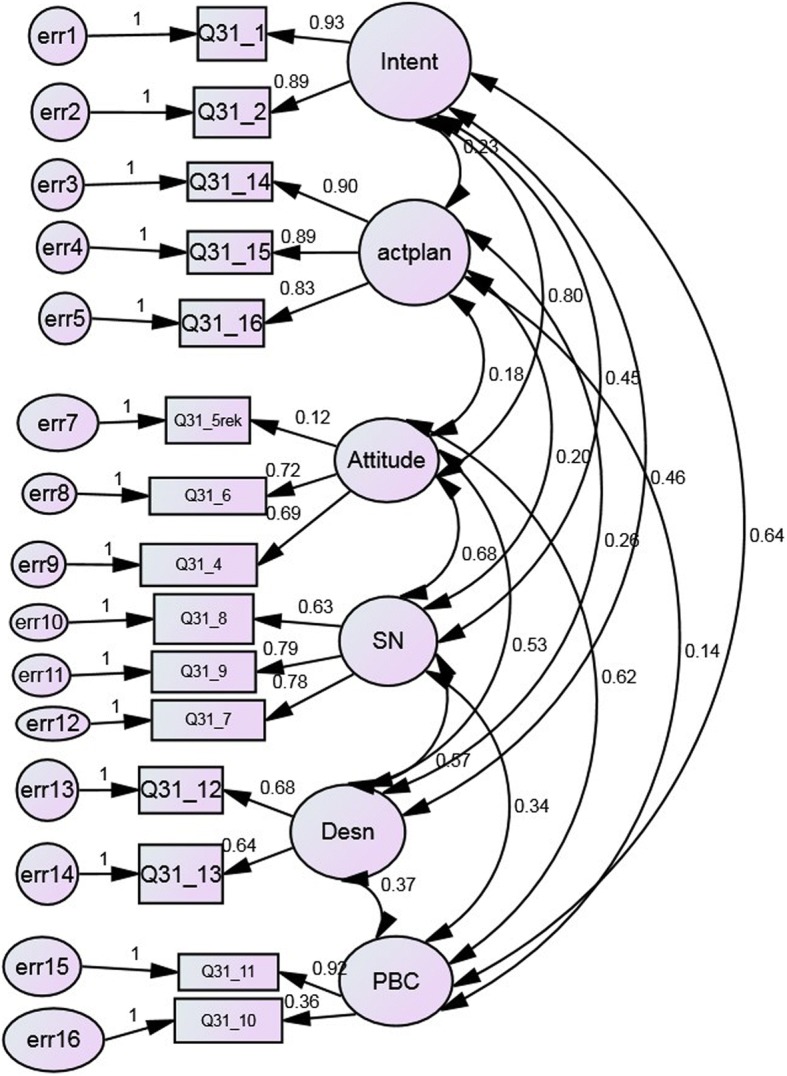
Modified 6-factor measurement model based on CFA

The default ML estimation with AMOS requires continuous multivariate normality of the observed indicator variables. As multivariate kurtosis represented by Mardia’s coefficient was below the recommended value of 3.0, it was not deemed necessary to bias correct estimates through bootstrapping [24]. According to the fit indices (CFI, RMSEA, AIC) employed, CFA indicated that an initially proposed correlated 6-factor model (attitudes, subjective norms, descriptive norms, perceived control, intention, action planning) was not an acceptable fit on any of the fit indices employed (CMIN (df) = 1457.6 (89), CFI = .925, RMSEA =0.058, AIC = 757.666). Inspection of modification indices indicated covariation between pairs of error terms, resulting from item overlap, or reflecting biases in responding such as “yea” saying or “no” saying. Attitude had a non-significant loading to one indicator (attend a dentist regularly is intolerable) which was removed from the model. Re-estimation of the 6-factor model gave acceptable fit (CMIN = (df) 655.666 (69), CFI = .96, RMSEA = .058, AIC = 757.666). As shown in Table 3, all factor loadings were in the expected direction and had significant regression weights with their related latent variables (C.R. > 1.96), indicating convergent validity. Most statistically significant items’ standardized regression weights were above 0.3, and thus in accordance with the threshold proposed [28]. Higher values of the indicators were associated with stronger (positive) attitudes, stronger subjective norms, descriptive norms, perceived behavioral control, intentions and action planning. The inter-factor correlations (correlations between the 6 latent variables) were below 0.85 indicating acceptable discriminant validity (< 0.85). Figure 1 depicts the modified 6- factor measurement model based on CFA.

**Table 3:** Standardized regression weights for the different components of the modified correlated 6-factor measure model including intention(INT), attitudes (ATT), subjective norms (SN), descriptive norms (DN), perceived behavioral control (PBC), action planning (AP)

Parameters	Observed variable (figure label)	Parameter estimate (factor loading)
INT	I intend to attend (Q31_1)	0.927 ***
	I have decided to attend (Q3_2)	0.890***
ATT	To attend is reasonable (Q31_4)	0.695***
	To attend is necessary (Q31_6)	0.715***
	To attend is an economic burden (Q31_5)	0.117***
SN	My parents want me to attend (Q31_7)	0.789***
	My friends want me to attend (Q31_8)	0.627***
	My dentist want me to attend (Q31_9)	0.792***
DN	My friends attend (Q31_12)	0.680***
	My parents attend (Q31_13)	0.642***
PBC	Its up to me whether to attend (Q31_10)	0.358***
	I am capable to attend (Q31_11)	0.916***
AP	I have made a detailed plan when (Q31_14)	0.901***
	I have made a detailed plan where (Q31_14)	0.895***
	I have made a detailed plan how (Q31_15)	0.832***

***p < 0.001

Gender specific modified correlated factor models indicated acceptable fit for males (CMIN 306, df 69, *p* < 0.000, CFI = 0.968, RMSEA = 0.056) as well as for females (CMIN 402,846, df = 69, *p* < 0.000, CFI = 0.964, RMSEA = 0.058). Multi-group analyses, testing for invariance across males and females simultaneously, revealed acceptable fit for the unconstrained model (CMIN = 709.689, df 138, *p* < 0.000, CFI = 0.966, RMSEA = 0.040) indicating configural invariance (equivalent factor structure). Compared to the unconstrained baseline model, a model with constrained measurement weights were statistically significant (CMIN 738.00 df147, *p* < 0.001, CFI = 0.964, RMSEA = 0.040). As indicated by the slightly increase in CMIN and decline in CFI values as compared to those in the unconstrained configural model, some variance in factor loadings could be expected across males and females. The difference ∆ CMIN =28.319, DF 9 was statistically significant at *p* < 0.001 indicating lack of metric invariance or at best partial invariance for the factor loadings.

### Structural equation model

**Fig. 2:**
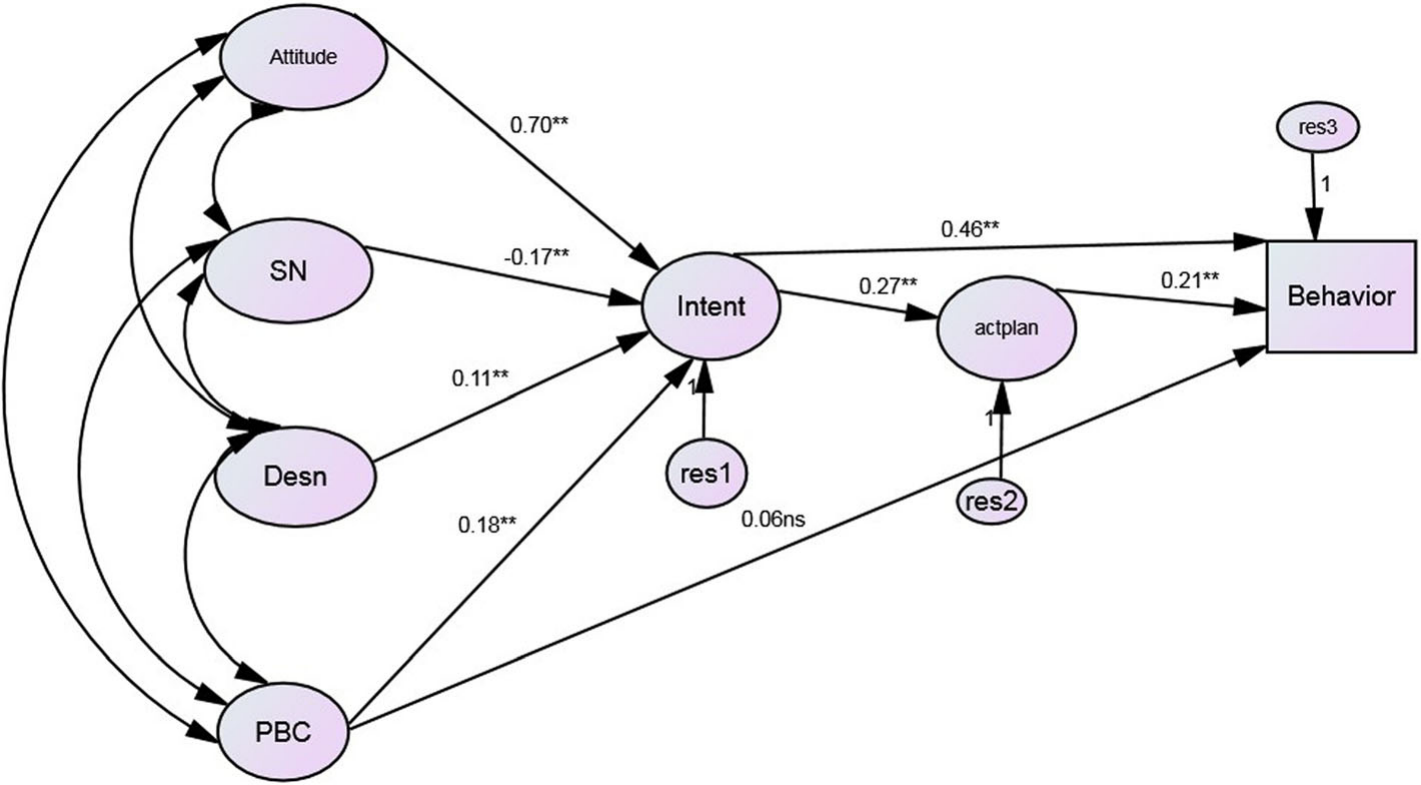
The augmented Theory of Planned Behavior structural equation model. For ease of interpretation only direct pathways are shown

Structural equation modelling, SEM, was conducted to estimate the fit of the augmented TPB model and the relationships among the latent constructs. The model with intention (INT), action planning (AP), and dental attendance predicted by attitudes (ATT), subjective norms (SN), descriptive norms (DN) and perceived behavioral control (PBC) was an acceptable fit to the data; CMIN 821.234 (85), *p* < 0.001, CFI = 0.959, RMSEA = 0.058 and AIC = 923,234). Direct paths from attitudes, subjective norms and descriptive norms on dental attendance behavior did not improve the fit of the model and none of those paths was statistically significant. Figure 2 depicts the direct effects for the augmented TPB model.

As hypothesized by the augmented TPB, stronger attitudes β = .70, *p* < 0.001, perceived behavioral control β = 0.18, *p* < 0.001 and descriptive norm β = .11, *p* < 0.001 were all linked to stronger intentions (Table 4). Subjective norm was negatively related to intention β = −-17, *p* < 0.001. Stronger intention was linked to stronger action planning β = .27, *p* < 0.001 and to more frequent dental attendance β =0.46, *p* < 0.001. Stronger PBC was also linked to more frequent dental attendance, however this path was not statistically significantly β =0.06, 0.03n.s. Stronger action planning was linked to more frequent dental attendance β = .19, *p* < 0.001. Attitudes, subjective norms, descriptive norms and perceived behavioral control accounted for 64% of the variance in intention, intention accounted for 7.6% of the variance in action planning and intention, action planning and perceived behavioral control accounted for 32% variance in dental attendance. Specific indirect effects were estimated by multiplying the direct effects of the variables involved in the total pathway. An indirect path from perceived behavioral control to behavior (β = 0.01) was as follows; Perceived behavioral control-intention (β = .18), intention-action planning (β = .27), action planning-behavior (β = .19). This indicates that the effect of perceived behavioral control on dental attendance was primarily through intention and action planning. An indirect path from intention to behavior (β =0.05) was as follows; intention-action planning (β = 0.27), action planning-behavior (β =0.19). The effect of intention on behavior was primarily a direct one.

**Table 4:** Significant direct standardized regression weights for the extended theory of planned behavior- Modified SEM model

	Standardized regression weight	% total effect
Direct standardized effects		
Intention-attitudes	.76 (.70)***	Intention: 64
Intention-subjective norms	.-19 (.-17)***	Action plan: 7.6
Intention-descriptive norm	.11 (.11)***	Behavior: 32
Intention-perceived control	.16 (.18)***	
Intention-Action plan	.27 (.27)***	
Action plan-behavior	.21 (.19)***	
Intention -Behavior	.51(.46)***	
Perceived control-behavior	.06 (.03)ns	
Indirect standardized effects		
Perceived control-intention-action plan - behavior	.01	
Intention-action plan -behavior	.05	

***p < 0.001

## Discussion

The present study examined, for the first time, the effect of motivational (intention) and volitional (action planning) factors upon regular dental attendance using a cross-sectional design, a structural equation modelling approach (SEM) and a representative sample of Norwegian adults 25–35 years of age. The benefit of SEM over other statistical procedures is its ability to test the hypothesized relationships among observed and latent variables in the TPB model completely and simultaneously. Structural equation modelling has gained considerable popularity and whilst modelling the TPB constructs as latent variables shows the ability to account for measurement errors, which may influence the relationships in the model [25, 26].

This study revealed that the proportion of dental attendance at least once a year amounted to 47.2% among 25–35 year old Norwegian adults. This prevalence rate is marginally lower and higher than those reported among 25-year-old Norwegians in 1997 (62%) and 2007 (44.6%), respectively, and deviates with figures from 2013 indicating that 63% of 20–39 year olds had visited a dentist within the previous year [6, 9]. Nevertheless, dental attendance rate is not satisfactory as long as 21% reported dental attendance frequency less than every second year.

In a first step, a modified correlated factor analytical model provided support for the factorial validity of a questionnaire supposed to measure intention, action planning, attitudes, subjective norms, perceived control and descriptive norms thus confirming construct validity of a modified 6-factor model (Fig. 1). Although a small and statistically significant *p*-value for the chi-square statistics indicated poor fit of the measurement model, by taking sample size into consideration, the comparative fit indices fulfilled the criteria for good fit [24, 26]. In the final model, all inter-factor correlations were below the threshold set to indicate poor discriminative validity [25–27]. Structural equation modelling in a second step showed that the augmented TPB model applied was a good fit to the data explaining large amounts of variation in intention and attendance behavior. In addition, multi-group analysis revealed that the structural part (configural invariance) of the model operated equivalently across males and females, although the factor loadings did not achieve invariance.

The present finings add to previous findings considering the ability of the augmented TPB to account for greater variance in intention and behavior than the TPB alone [28, 29]. The explained variance in intention (64%) and behavior (32%) was higher than that commonly reported in meta-analyses of the TPB, being in the range of 40–49% for intention and 26–36% regarding actual behavior [14, 15]. Thus, the results revealed direct statistically significant pathways to intention from attitude (β = .70), subjective norm (β = .-.17), PBC (β = .18), and descriptive norm (β = .11). These findings support the hypothesis that TPB augmented with descriptive norm would predict behavior indirectly through behavioral intention. A direct path from descriptive norm to intention suggests that young adults are guided by what others do regarding their dental visiting behavior. Meta analytical reviews of health related behaviors have also revealed that descriptive norm adds to the prediction of intention independent of the TPB constructs [30, 31]. Nevertheless, attitudes were the strongest motivational determinant implying that younger adults’ decision to attend a dentist on a regular basis was almost entirely based on anticipated benefits of that behavior but also on social norms (subjective norm and descriptive norm) and considerations of potential obstacles (perceived behavioral control), in that order. The finding that attitudes and perceived behavioral control are predictive of intended dental attendance is in line with other studies predicting decisions to utilize health care services [1, 16]. These findings imply that reduced perceived control due to barriers, such as for instance dental anxiety and fear, would reduce intention and actual use of dental health care services among younger Norwegian adults. Unexpectedly, the direction of the path from subjective norms to intention was negative implying that higher perceived social approval for dental attendance result in lower motivation for that behavior. Although speculative, the construct of psychological reactance may offer an explanation to this uncommon finding as psychological reactance effects in health related behavior have been observed previously in various domains [20]. In practical terms, however, interventions targeting young adults’ dental attendance behavior might usefully focus on informed awareness of the positive oral health consequences following this behavior accompanied with strategies such as modeling and group performance. Educational messages aimed at increasing young adults’ regular dental attendance could highlight the prevalence of dental attendance among the youth in the community. If young adults get a sense that everybody at their own age is attending on a regular basis, they might be encourage to abandon their non-attendance.

Intention was by far the strongest predictor of dental attendance (β = .46), whereas action planning came second (β = .21). This supports the hypothesis that action planning contributes to the prediction of dental attendance over and above the effect of intention whereas the association between intention and behavior was partly mediated through action planning. Forming action plans considering when, where and how to act facilitates behavioral action by setting situational cues that activate cognitive processes needed to execute the behavior and highlights that intenders may benefit from formulating plans to engage in regular dental attendance [28, 29]. Previous studies have revealed that action planning contributes to the prediction of health service utilization, such as cervical cancer screening [18, 20]. Schutz et al. [28] and Åstrøm [19] examined subsequent flossing in the context of social cognition theory and found that action planning was a significant predictor of actual flossing alongside intentions and previous flossing. Consistent with those studies, but at odds with others [18, 19, 28], the present one found action planning to be a significant predictor of dental attendance. Inconsistent with TPB, perceived behavioral control did not emerge as a significant predictor of dental attendance. This accords, however, with a meta-analysis by Cooke and French [31], where perceived control was an unimportant predictor of screening behavior. Thus, attending a dentist on regular basis seem to be under complete volitional control by younger adults in Norway, who do not require particular resources, opportunities and technical skills for performance [13].

This study should be interpreted within the context of its strengths and limitations. The evidence provided from a large population based study that dental attendance is strongly associated with action planning and intention, which in turn is associated with attitudes, subjective norms, descriptive norms and perceived behavioral control identifies targets for informing dental health care interventions among young adults in Norway. A limitation of the present study was the use of self-reported dental attendance that might be biased by social desirability bias resulting in over reporting as compared with medical records. Evidence suggests that the validity of self-reported use of dental services ranges from poor to excellent, depending on service type [32]. Moreover, the dental attendance question was adopted from previously tested measures and it is reasonable to assume that it was sufficiently simple and unambiguous to achieve a satisfactory degree of reliability. Another weakness associated with the present cross-sectional study, as with most population based electronically administered surveys, is the relatively low response rate. Comparison of sex, age and educational level distributions among participants with the corresponding figures in the population provided by official statistics showed a similar age- and educational level distribution but a moderately different gender distribution that most probably did not affect the generalizability of the findings presented. A further weakness is the use of a cross –sectional design, thus violating Ajzen’s recommended longitudinal design for the original TPB model [13]. Measuring intention to attend dentists and self-reported dental attendance in one point in time might have resulted in an unrealistic high explained variance of behavior since the intention behavior gap widens the longer the time interval between intention and behavior [13]. Further studies should incorporate subsequent measures of behavior or use information from dental records to validate the self-report measure utilized in the present study.

## Conclusions

The presents study is the first large nationally representative population based study analyzing younger adults’ dental attendance behavior within the context of an augmented TPB model and using a structural equation modeling approach. The present findings support the utility of the TPB, the expanded normative component and the construct of action planning in predicting younger adults’ intended and self-reported dental attendance. Interventions targeting young adults’ dental attendance behavior might usefully focus on positive consequences following this behavior accompanied with modeling and group performance.

## Additional file

Additional file 1: Questionnaire in English language version translated from Norwegian. (DOCX 18 kb)

## Abbreviations

AIC: Aikaikes
AP: Action planning
ATT: Attitudes
CFA: Confirmatory factor analysis
CFI: Comparative fit index
CMIN: minimum discrepancy
CR: Critical ratio
DN: Descriptive norms
INT: Intention
MI: Modification indices
ML: Maximum likelihood
PBC: Perceived behavioral control
RMSEA: root mean square error of approximation
SEM: Structural Equation Modeling
SN: subjective norms
SPSS: Statistical Packages for Social Sciences
TPB: Theory of planned behavior
