# Supplementary material for: Applying the theory of planned behavior to self-report dental attendance in Norwegian adults through structural equation modelling approach
Source: BMC Oral Health. 2018 May 31;18:95. doi: 10.1186/s12903-018-0558-7 (PMC5984321; doi:10.1186/s12903-018-0558-7)
Supplement: Supplementary file 1 — Questionnaire in English language version translated from Norwegian. (DOCX 18 kb) [file 12903_2018_558_MOESM1_ESM.docx]

Begynn her:

1. ***I am***

□ Male

□ female

1. ***How old are you?____________________________***
2. ***Your highest education?***

□ No education

□ Primary education

□ Secondary education

□ Bachelor/college

□ University /high school

1. What is your current civil status?

□ Married

□ Not married

□ Divorsed

□ Widow

1. In what country were you borne?

□ Norway

□ Another Nordic country

□ A country outside the Nordic countries

1. How large was your household income (in thousand Norwegian kroner)

□0-99

□100-149

□150-199

□200-249

□250-299

□300-399

□400-499

□500-599

□600 og mer

**40. English translation of the questionnaire assessing the constructs of the augmented model of the Theory of Planned Behavior**

|  | Strongly agree | Partly agree | Agree nor disagree | Partly disagree | Strongly disagree |
| --- | --- | --- | --- | --- | --- |
| I intend to attend a dentist regularly |  |  |  |  |  |
| I have made a decision to attend a dentist regularly |  |  |  |  |  |
| To attend a dentist regularly in the future is intolerable for me |  |  |  |  |  |
| To attend a dentist regularly in the future is reasonable for me |  |  |  |  |  |
| To attend a dentist regularly in the future is necessary for me |  |  |  |  |  |
| To attend a dentist regularly in the future is an economic burden for me |  |  |  |  |  |
| My parents want me to attend a dentist regularly in the future |  |  |  |  |  |
| My partner want me to attend a dentist regularly in the future |  |  |  |  |  |
| My dentist want me to attend a dentist regularly in the future |  |  |  |  |  |
| Its up to me whether or nor I attend a dentist regularly in the future |  |  |  |  |  |
| I am capable to attend a dentist regularly in the future |  |  |  |  |  |
| My friends attend a dentist regularly in the future |  |  |  |  |  |
| My parents attend a dentist regularly in the future |  |  |  |  |  |
| I have made a detailed plan ragrding when to attend a dentist i the future |  |  |  |  |  |
| I have made a detailed paln where to attend a dentist in the future |  |  |  |  |  |
| I have made a detailed plan how to attend a dentist in the future |  |  |  |  |  |
